# The stroke meta-metric, Defect-Free Care, was maintained year-over-year within the Florida stroke registry during the COVID-19 pandemic

**DOI:** 10.1016/j.jstrokecerebrovasdis.2024.108179

**Published:** 2024-12-06

**Authors:** David Z. Rose, Lili Zhou, Karlon H. Johnson, Charles Schutt, Daniel M. Reyes de Jesus, Hannah Gardener, Carolina M. Gutierrez, Dianne Foster, Angus Jameson, Sebastian Koch, Hao Ying, Ayham Alkhachroum, Jose G. Romano, Tatjana Rundek, Negar Asdaghi

**Affiliations:** a University of South Florida Morsani College of Medicine, Tampa, FL USA; b University of Miami Miller School of Medicine, Miami, FL USA; c Sacred Heart Hospital, Ascension Health, Pensacola, FL, USA; d Hospital Menonita, Caguas, PR, USA; e American Heart Association, Southeast Region, Atlanta, GA, USA; f Pinellas County Emergency Medical Services, FL, USA

**Keywords:** Ischemic stroke, COVID19, Pandemic, Outcomes, Metrics

## Abstract

**Background:**

Resource allocation for stroke care was impacted worldwide by the Coronavirus 2019 (COVID19) pandemic. Regionally, worsened stroke outcomes varied, however comparative year-over-year in-hospital performance metrics from the pandemic are unreported. Therefore, within the large Florida Stroke Registry (FSR), we assessed the pandemic’s effects upon the American Heart Association (AHA) Get With The Guidelines (GWTG) ischemic stroke metrics and the meta-metric, Defect-Free Care (DFC).

**Methods:**

From March 2017 to February 2021, FSR collected 146,593 patients with a diagnosis of ischemic stroke or TIA (31,940 between 2017–2018; 35,086 between 2018–2019; 39,722 between 2019–2020; 39,845 between 2020–2021). FSR evaluated DFC, intravenous thrombolytic (IVT) use, endovascular therapy (EVT) use, and Door-To-Needle (DTN), Door-To-Computed Tomography (DTCT), and Door-To-Puncture (DTP) times.

**Results:**

Pre-pandemic versus pandemic stroke patients’ demographics were similar (49.0 % vs. 48.6 % female, age 70.7±14.3 vs. 70.5±14.1 years, 64.0 % vs. 65.3 % white, 18.6 % vs. 18.8 % black, 17.4 % vs. 16.0 % Hispanic). Pandemic strokes, versus the immediate year pre-pandemic, were significantly more severe (median NIHSS 4 [IQR 8] vs. 3 [7]), utilized emergency medical services more (59.2 % vs. 57.6 %) and were more likely to receive EVT (8.0 % vs. 7.0 %). IVT use, and DTCT and DTP times were unchanged. The meta-metric DFC improved year-over-year, albeit slower during the pandemic [2017/18–2018/19: 70.7 % to 74.9 % (+4.2 %); 2018/19–2019/20: 74.9 % to 82.1 % (+7.2 %); 2019/20–2020/21: 82.1 % to 85.2 % (+3.1 %)].

**Conclusions:**

Despite pandemic challenges, the stroke meta-metric DFC improved, albeit more slowly than pre-pandemic years. In this large statewide registry, pandemic patients received EVT more, potentially due to more severe stroke presentations. Stroke care infrastructure preparedness for future pandemics is warranted.

## Introduction

Since the official end of the Coronavirus disease 2019 (COVID19) pandemic, multiple studies reported that COVID19 altered several aspects of healthcare delivery and resource allocation for time-sensitive illnesses such as stroke. The pleiotropic effect of the virus stretched beyond its pulmonary role: the triage and treatment of stroke patients were impacted on a global scale. Also, many diverse neurological complications and sequelae of COVID19 infection upon the central nervous system were seen,^1^ associated with endotheliopathy and systemic inflammatory responses.^2^ Specifically, ischemic stroke patients with COVID19 often experienced worse outcomes and developed more frequent and severe/life-threatening complications.^3–5^

In 2020, at the beginning of the pandemic, observational studies noted a paradoxical decrease in stroke census at many hospitals.^4,6–10^ This was ostensibly related to social distancing, quarantines, and “Stay at Home” lockdown mandates, regrettably misapplied to patients with mild stroke symptoms or transient ischemic attack (TIA). Overtly disabled stroke patients, discovered by family neurologically devastated, were still taken to emergency rooms, however less ill patients avoided hospitals because of so-called “COVID-ophobia” – a nosocomephobia/fear of contracting coronavirus while hospitalized.^4^ Before the vaccine was available, COVID19 mortality exceeded stroke mortality^11^ which dropped to number 6 on the list of deadly diseases as COVID19 assumed the 5^th^ position. As COVID19 waned, stroke has retaken the number 5 spot.

Because attention focused upon COVID19, and its unprecedented challenges upon the healthcare system, there may have been a resultant decrease in adherence to stroke quality measures, such as the American Heart Association (AHA) Get with the Guidelines (GWTG) metrics. Indeed, stroke reperfusion therapies, intravenous thrombolytic (IVT) and endovascular therapy (EVT), were delayed by the pandemic and worsened outcomes in several reports.^12–14^ However, some manuscripts on this topic were retracted by journals because of lack of written consent from AHA to use GWTG dataset and hyper-expedited peer-reviews (intended to deliver the latest pandemic discoveries quickly) resulted in errors in methodology and conclusions.^15^ Therefore, an unmet need still exists to comprehensively and accurately assess pandemic GWTG stroke measures and the meta-metric, Defect-Free Care (DFC), which includes 7 domains reflective of optimal stroke care. The rationale for analyzing DFC is threefold: first, DFC diversifies multiple metrics of overall stroke care rather than focusing on one or two metrics in which one health system may excel or not; secondly, DFC as a composite variable is an indicator of whether appropriate interventions are performed properly together for statistically superior hospital-wide performance; and third, prior publications have shown that quality improvement (QI) programs targeting areas of suboptimal DFC performance may reduce racial-ethnic, socio-economic (and other) disparities.^16^ To assess hospital care systems aside from only DFC, we also included items such as severity of stroke and timing of treatment, which provides a real-world backdrop in which pandemic stroke care can be systematically reviewed.

We sought to study this within the large, statewide, Florida Stroke Registry (FSR), and identify differences in year-over-year inpatient stroke care trends. Our hypothesis was that DFC would continue to improve as it had done in years past, even through pandemic challenges. However, a rejection of this hypothesis would imply that pandemic-induced system-wide effects may have delayed critical, time-sensitive stroke triage and management.

## Methods

### Data availability statement

The FSR uses data from Get With the Guideline-Stroke (GWTG-S), which is an in-hospital program that promotes consistent adherence to the latest scientific treatment guidelines. GWTG-S is collected primarily for quality improvement and achieving measurable patient outcome metrics; data-sharing agreements require an application process for other researchers to access data. Research proposals can be submitted at www.heart.org/qualityresearch and will be considered by the GWTG-S and the FSR publications committee upon request.

### Registry information and study population

The FSR is a registry that began in 2012 collecting data on patients with ischemic stroke, intracerebral hemorrhage, subarachnoid hemorrhage, and TIA from GWTG-Stroke participating hospitals in Florida.^16^ Just before the COVID19 pandemic, 80 hospitals participated in FSR, and now, over 170 have joined. Stroke care metrics were compared in patients enrolled during the COVID19 pandemic (March 2020 to February 2021) to those enrolled in the immediate pre-pandemic year (March 2019 to February 2020) and as well versus the previous three years (March 2017 to February 2020). Patients with intracerebral and subarachnoid hemorrhage were excluded from this particular study in order to focus on DFC in ischemic stroke only. Multiple variables have been collected since inception, which include race and ethnic categories, as previously described^16^ categorized as FL-White (non-Hispanic White residing in Florida), FL-Black (non-Hispanic Black residing in Florida), and FL-Hispanic (Hispanic of any race residing in Florida). Metrics evaluated included utilization of intravenous thrombolytic (IVT), endovascular therapy (EVT), Door-To-Needle (DTN), Door-To-Puncture (DTP), Door-To-Computed Tomography (DTCT) times, and overall DFC as reviewed below. This study is approved by the University of Miami’s institutional review board. Institutional ethics approval was approved for each GWTG-Stroke participating hospital to enroll cases in the FSR without requiring individual patient consent under the common rule or a waiver of authorization and exemption from subsequent review by their institutional review board.

### Defect-free care (DFC)

As reported in prior publications,^16^ DFC includes 7 domains reflective of optimal stroke care: 1. IVT arrive by 3.5 hours and treatment by 4.5 hours; 2. early anti-thrombotic use; 3. venous thromboembolism (VTE) prophylaxis; 4. anti-thrombotic prescription at discharge; 5. anticoagulation for atrial fibrillation in patients without contraindication; 6. smoking cessation discussion documentation; and 7. high-intensity statin use in patients without contraindication. As a measure of comprehensive care including patient education rather than direct treatment alone, DFC has a track-record of use and utility; prior research suggests that disparities in stroke care can be identified in hospitals with suboptimal DFC performance, for which QI programs can be designed to mitigate these disparities.^16^

### Standardization

To enhance generalizability and applicability of the data, and avoid analyzing DFC in isolation, we also included other time-critical stroke metrics: DTCT, DTN, and DTP times (measured in minutes) – items that were data-captured by all participating FSR hospitals. These standardized performance metrics determine efficiency of diagnostic delivery and stroke treatment, whether IVT or EVT or both. Onset-to-arrival time is defined as the time from onset of stroke symptoms (or time that patient was last known well) to the time of arrival to the hospital. DTCT is defined as the time from triage (ED arrival) to initial neuroimaging. DTN is defined as the time from stroke patient arrival to initiation of IVT bolus. DTP is the time from arrival to arterial puncture for intra-arterial therapy in the endovascular suite.

The outcome metrics of IVT and EVT utilization were determined by binary status of receipt or non-receipt of each type of therapy for each patient (yes/no). Median National Institute of Health Stroke Scale (NIHSS) scores were measured to determine stroke severity among each group of patients, with higher scores indicating greater stroke severity. DFC rates assess the overall delivery of fully appropriate interventions for each patient at all FSR hospitals.

### Statistical analysis

Continuous variables were compared using means with standard deviation. For continuous variables with non-Normal distributions, we used medians with interquartile ranges. Categorical variables were calculated using percentage frequencies. We used an ANOVA test to compare average DTP, DTN, and DTCT in pandemic vs. non-pandemic stroke patients. We set these tests at a significance threshold of p < 0.05. For comparison of IVT and EVT utilization between patient groups, we used chi square test. We set a significance threshold of p < 0.05. SAS 9.4 was used to conduct all statistical analyses.

## Results

From March 2017 to February 2021, FSR identified 146,593 patients with a diagnosis of ischemic stroke or TIA (31,940 between March 2017 to February 2018; 35,086 between March 2018 to February 2019; 39,722 between March 2019 to February 2020; and 39,845 between March 2020 to February 2021). Stroke patients who were treated during the three years (March 2017 to February 2020) preceding the COVID19 pandemic (n=106,748, 49.0 % female, mean age 70.7±14.3 years, 64.0 % white, 18.6 % black, 17.4 % Hispanic) had similar demographics to those during the COVID19 pandemic (n=39,845, 48.6 % female, mean age 70.5±14.1 years, 65.3 % white, 18.8 % black, 16.0 % Hispanic), as seen in Table 1. When compared to the immediate pre-pandemic year (March 2019 to February 2020), pandemic strokes were more severe (median NIHSS 4 [IQR 8] vs 3 [8], p<0.0001), and utilized emergency medical services more (59.2 % vs. 57.6 %, p<0.0001). However, this significance was not sustained when combining the three pre-pandemic years together (median NIHSS 4 [8] vs 4 [8], p=0.22) and (58.7 % vs. 59.2 %, p=0.07) (Table 2). Additionally, the prevalence of acute ischemic stroke (AIS) remained significantly higher than the prevalence of TIA when comparing pre-pandemic to pandemic periods (91.5 % vs 92.8 % AIS in pre-pandemic vs during pandemic, p<0.0001).

Overall, compared to pre-pandemic years, other stroke metrics observed during the pandemic did not change: IVT use (13.3 % vs. 12.9 %, p=0.65), onset-to-arrival time (264 [709] vs. 224 [630] minutes, p=0.96), DTN time (37 [21] vs. 38 [23] minutes, p=0.07), DTP time (84 [62] vs. 81 [66] minutes, p=0.80), and DTCT time (22 [52] vs. 24 [57] minutes, p=0.37).

**Graph 1.**
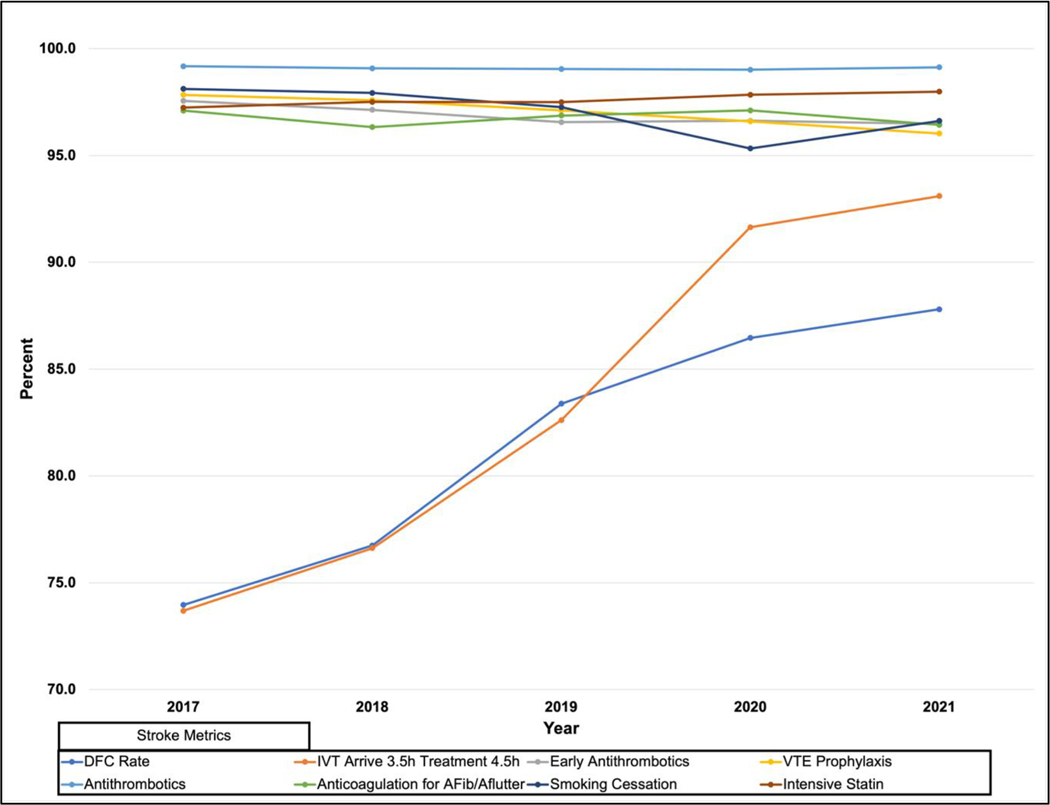
The annual trends of the Florida Stroke Registry (FSR) Defect-free care (DFC) rate and its seven domains from 2017–2021. DFC improved year-over-year. However, this rate slowed down (lesser slope) during the pandemic (2020–2021).

**Graph 2.**
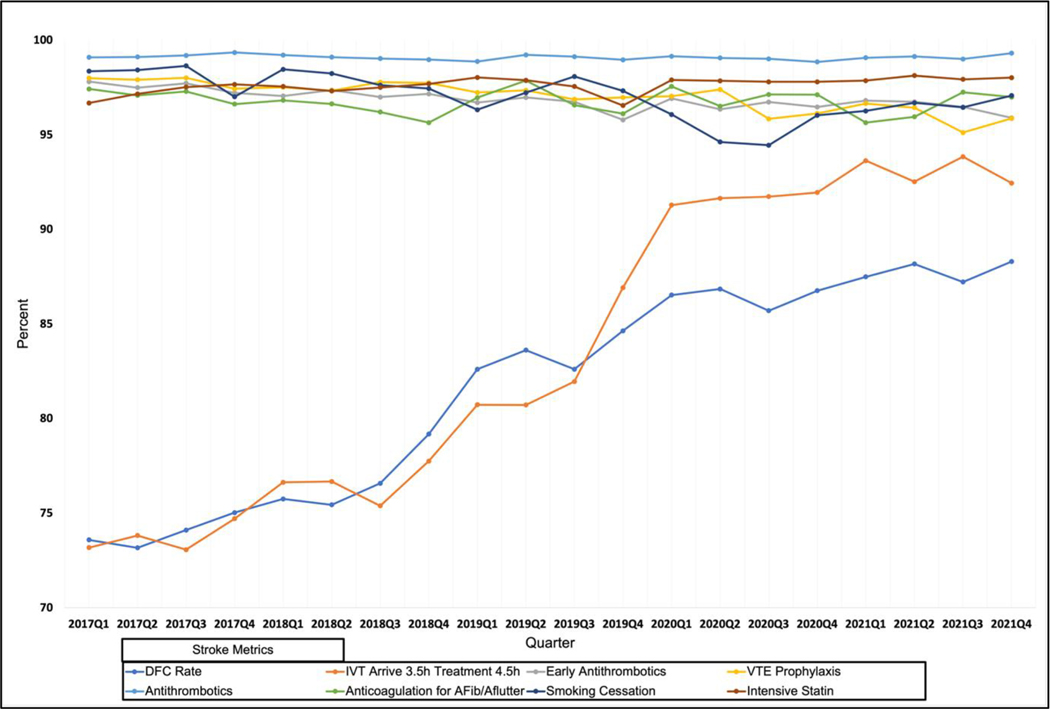
The quarterly trends of the Florida Stroke Registry (FSR) Defect-free care (DFC) rate and its seven domains’ from 2017 Quarter 1 (Q1) to 2021 Quarter 4 (Q4). The DFC rate improved gradually by quarter but slowed during the pandemic quarters, especially 2020 Q2-Q4.

However, pandemic stroke patients were more likely to receive EVT than pre-pandemic patients (8.0 % vs. 7.0 %, p<0.0001). Furthermore, DFC improved significantly when comparing pre-pandemic years to the pandemic (76.3 % vs. 85.2 %, p<.0001). Year-over-year DFC improved (Supplemental Tables 1 and 2; Graph 1 and 2), however, at a slower rate during the pandemic period than each prior year of improvement: from March 2017/February 2018 to March 2018/February 2019, 70.7 % to 74.9 % (+4.2 %); from March 2018/February 2019 to March 2019/February 2020: 74.9 % to 82.1 % (+7.2 %); but from March 2019/February 2020 to March 2020/February 2021, only from 82.1 % to 85.2 % (+3.1 %). Supplemental Table 3 shows that improvements in DFC from pre-pandemic to pandemic were consistent regardless of mild/moderate/severe NIHSS (p<.0001).

## Discussion

In this large, statewide, hospital-based registry, our hypothesis was accepted: stroke care, as measured by DFC, continued to improve as it had done in years past, even through the panoply of pandemic challenges. With statistically similar demographics among over 100,000 stroke patients in the pre-pandemic timeframe and nearly 40,000 stroke patients during the pandemic, we found that patients treated for stroke during the COVID19 pandemic in the state of Florida, as assessed with GWTG metrics, such as IVT use, onset-to-arrival, DTP, and DTCT times were all unchanged during the pandemic, compared to prior years. Compared to the immediate preceding year, pandemic stroke patients presented sicker, utilized emergency medical services more and were more likely to receive EVT. Reasons for this include: “Stay at Home” orders convinced TIA/minor stroke patients to avoid hospitals (augmenting the ratio of severe strokes requiring EVT), COVID19 hypercoagulability may have resulted in more large vessel occlusions (LVO), and EVT acceptance and technical advancements in hyperacute clot retrieval progressed irrespective of the pandemic.

This data, prima facie, may seem counter to reports of pandemic-induced disruptions to systems of care for non-COVID19 patients, which were published as early as March 2020, claiming overwhelmed healthcare resources impacted ischemic and hemorrhagic stroke triage and treatment.^17^ Mass screening for COVID19 postponed urgent brain scans, neurologic intensive-care units hosted COVID19 patients in isolation, sending stroke patients elsewhere into the hospital, and “Stay at Home” orders encouraged abbreviated hospitalizations, outpatient workup, and expedited discharges.^4^

The ultimate concern was that so many healthcare disruptions by COVID19 may have stunted – or even reversed – years of DFC progress. However, we found that DFC did not worsen during the pandemic, it actually continued to improve. The strong statewide effort and FSR priority to maintain DFC, securing all 7 DFC metrics at or near 100 % has been the ultimate goal over the years since FSR inception in 2012. Grassroots programs, annual in-state conferences, multiple journal publications, and constant communications and reminders encouraging best practices, likely all improved year-over-year DFC rate. Nevertheless, the rate of DFC increase (acceleration) occurred more slowly during the pandemic than in prior years: the rise of 3.1 %, while a positive result, was significantly less positive (represented by a lesser slope) versus 7.2 % from the previous year, and 4.2 % from the year before that. This may be related to COVID19 challenges as listed above, or rather from a ceiling-effect that may limit improvements as each metric approaches the absolute highest possible adherence of 100 % maximum. However, collectively, FSR hospitals had not yet actualized the full-100 % DFC yet. Therefore, at least part of this slower DFC rise may be due to pandemic-related items: physician burnout, sequestered resources, and unmeasured or undocumented stroke care metrics. Regardless, even a smaller DFC rise is superior to a reversal or decline, and indicates that a high quality of stroke care can be maintained during a pandemic. Whether this deceleration persists, or if the slope returns to its previous rate of rise, remains to be seen.

Other stroke registries have reported on surrogate biomarkers to assess the effects of care during the pandemic. A cross-sectional study of stroke hospitalizations in 2020 revealed an 11.5 % decrease in stroke admissions, 13.2 % drop in IVT and 11.9 % drop in IVT transfers.^18^ A survey of physicians across several countries reported a 33 % drop in stroke admissions and 25 % fewer EVT procedures.^19^ In Japan, impaired consciousness scores were reportedly more severe during the pandemic^20^ and in New York City, early pandemic stroke patients had higher median admission NIHSS without a difference in onset of symptoms to arrival, DTCT, DTN, or DTP.^11^ Another New York study reported a stroke census volume drop by 49.5 % during the pandemic with more patients presenting after 24 hours from last known well, and no significant difference in EVT use.^21^ A study across several U.S. states identified more disabled patients (NIHSS>14) during the pandemic, but similar LVO and IVT use versus a pre-pandemic period.^22^ A study across several western countries found increased DTP.^23^

Such variance is multifactorial, as geographical differences in triage and severity of both stroke and of COVID19, which peaked at different times across the globe, resulted in data being reported irregularly, typically after COVID19 peaks, and mostly before the pandemic ended. Also, stroke registries are organized differently around the country and world; many do not capture stroke metrics with GWTG. While smaller observational studies offer insight into hospital-specific, pandemic-induced, stroke care delays, larger international studies represent a mix of metrics and carry less generalizability and utility for future pre-paredness. Our FSR study – in essence a hybrid of the two approaches above – is ideal for assessment of a meta-metric, DFC, which improved despite increasing stroke admissions each year, even during the pandemic. This differs from other reports: in Switzerland, stroke admissions dropped by 22 % during the pandemic compared to 2018–2019 however delivery and quality of stroke care were maintained.^24^

Some studies claimed that the pandemic decreased overall stroke services, neuroimaging scans and telehealth consultations: a report from Spain found a 25 % reduction in stroke cases, a 72 % reduction in telehealth stroke requests, and increases in onset-to-door and DTN times.^25^ A retrospective study of suspected stroke patients across U.S. hospitals, using the Viz.AI neuroimaging platform, found a significant 22.8 % decline in computed tomography angiography (CTA) scans, and 26.1 % decrease in computed tomography perfusion (CTP) scans, with fewer comprehensive stroke center hospitalizations (18.8 % vs. 11.0 %).^26^ A study of the Rapid-AI neuroimaging platform revealed similar results in suspected stroke patients at U.S. hospitals: a 39 % drop in scans, especially in patients with CTP volumes ≤15ml.^27^

The common thread among all these reports in the literature is that when the pandemic hit, a shockwave of disruptions in healthcare delivery hit immediately, however as it progressed, hospitals adapted, shifting resources and personnel to retain the ability to meet the critical-care needs of the pandemic and simultaneously the urgent demands of stroke patients. This is congruent with the FSR’s continuously improving DFC metrics.

FSR is supported by the State of Florida, including legislative backup, designated state appropriations, and has divided the state into regional stroke-specific coalitions, which access shared data dashboards that track performance metrics online. By reviewing, and benchmarking regional performance at annual meetings, optimization of DFC occurs across all stakeholder hospitals. Of note, during the time in which this study was performed, 87 % of all FSR hospitals were nationally certified stroke centers, and the remaining hospitals were in the process of becoming certified. Regardless of certification status, all participating FSR hospitals must participate in GWTG in order to collect data for the meta-metric DFC. Community outreach is also a hallmark of FSR, providing stroke awareness materials in English, Spanish and Haitian Creole, public service announcements posted on signs at bus/train stands, and updates on social media and radio. These statewide programs may have led to patient buy-in for the DFC metrics as well, inadvertently. Another factor that may have led to the lack of decline in DFC could be related to a relatively less severe and shorter COVID19 shutdown period in Florida compared to other states; the stay-at-home executive order was enforced in Florida on April 1, 2020, and lifted in three phases starting as early as May 4, 2020. Theoretically, other states in which longer or more stringent stay-at-home orders were enforced may have experienced delays in stroke care and less DFC.

Although the COVID19 pandemic has officially ended, vigilance is key to preparedness for the impact of future pandemics upon stroke care. Areas for improvement include pre-hospital stroke triage,^28^ ambulance times,^29^ pre-identified secure and centralized stroke care centers during national emergencies, and stronger encouragement of the public to utilize stroke care centers in the hyperacute phase of stroke symptoms.^28^

### Limitations

Our study has limitations, being a secondary analysis of data from FSR, which has been utilizing the GWTG-Stroke registry from participating hospitals in Florida since 2012; although more hospitals continue to join FSR every year, this is a continuously increasing number, and hence the study is not fully generalizable. Secondly, the breadth and depth of FSR includes stroke-ready hospitals in small, rural communities as well as comprehensive stroke centers in large metropolitan cities, each facing pandemic peaks at various timepoints and within diverse healthcare system organizations. Thirdly, as the COVID19 pandemic was an unexpected global event, this study was not a primary analysis based on an *a priori* hypothesis, but rather a secondary analysis post-hoc. Fourth, this manuscript has “participation bias” in which newly participating hospitals are essentially measured against hospitals that have been involved with the FSR since inception years ago. A sensitivity analysis would diminish this bias, by comparing DFC only from the 80 hospitals participating in FSR at the beginning and also at the end of the pandemic; however the aim of this project was not to see how 80 hospitals fared during the pandemic, but rather to see how FSR continued to manage and adapt during the pandemic – and how all 170 hospitals, newer and older, complied with DFC metrics during a challenging time.

Lastly, our results indicate that despite healthcare delivery challenges imposed by COVID19, a high overall quality of stroke care in Florida hospitals was maintained with an improving DFC rate during the pandemic. Nevertheless, the slope of DFC improvement was less than in previous years, suggesting that the pandemic did have a somewhat negative consequence. However, we cannot exclude the possibility of this representing a ceiling effect, as DFC approaches 100%, or another (unknown) etiology that lowered the DFC rise that year alone. Hence, future research can evaluate later outcomes, years post-discharge and compare pre-pandemic and pandemic DFC to post-pandemic DFC.

## Conclusion

Our large, statewide, registry-based study found that compared to pre-pandemic care, stroke patients treated during the COVID19 pandemic presented sicker and were more likely to receive EVT. Despite many healthcare delivery challenges imposed by the pandemic, we found no change in DTCT times, IVT use, or DTN times. DFC continued to improve during the pandemic, albeit more slowly, which may be confounded by a ceiling-effect of the success of the registry itself. The sustained maintenance of quality stroke care by the FSR during this disruptive public health crisis serves as a testament for the adaptability of regional stroke-based coalitions. Further evaluation of stroke care infrastructure preparedness for future pandemics is warranted.

## Grant Support:

## Supplementary Material

MMC3

MMC1

MMC2

## Figures and Tables

**Table 1:** FSR Year-Over-Year Cohorts listed by demographics and stroke metrics.

	Year 1 (Mar 2017 – Feb 2018) n=31,940	Year 2 (Mar 2018 – Feb 2019) n=35,086	Year 3 (Mar 2019 – Feb 2020) n=39,722	Year 4 (Mar 2020 – Feb 2021) n=39,845	

Demographics					
Male (%)	50.7	51.0	51.3	51.4	
Female (%)	49.3	49.0	48.7	48.6	
Mean Age	70.9	70.7	70.6	70.47	
[Years (+SE)]	(14.4)	(14.2)	(14.3)	(14.1)	
Non-Hispanic	64.6	64.2	63.4	65.3	
White (%)					
Non-Hispanic	17.7	18.7	19.2	18.8	
Black (%)					
Hispanic (%)	17.8	17.1	17.5	16.0	
Stroke Metrics	**p-value**				
NIHSS Median	4 (9)	4 (8)	3 (8)	4 (8)	<0.0001
(IQR)					
Median Onset-to-Arrival	204 (576)	225 (629)	240 (675)	264 (709)	0.4084
Time [Minutes (IQR)]					
Arrive via	59.9	58.8	57.6	59.2	<0.0001
Emergency Medical Services (%)					
Utilization of IVT (%)	12.0	13.2	13.4	13.3	<0.0001
Utilization of EVT (%)	6.2	7.4	7.4	8.0	<0.0001
Median DTP	78 (62.5)	82 (73)	82 (65)	84 (62)	0.1128
Time [Minutes (IQR)]					
Median DTN	40 (24)	38 (23)	38 (23)	37 (21)	0.0618
Time [Minutes (IQR)]					
Median DTCT	26 (62)	23 (55)	23 (55)	22 (52)	0.2088
Time [Minutes (IQR)]					
DFC Rate (%)	70.7	74.9	82.1	85.2	<0.0001

**Table 2:** FSR Pre-pandemic vs. pandemic cohorts demographics and stroke metrics.

	Pre-pandemic	Pandemic	

	Mar 2017 - Feb 2020 (n=106,748)	Mar 2020 - Feb 2021 (n=39,845)	
Demographics			
Male (%)	50.99	51.38	
Female (%)	48.99	48.62	
Mean Age [yrs (+SE)]	70.74 (14.30)	70.47 (14.09)	
Non-Hispanic White (%)	64.01	65.25	
Non-Hispanic Black (%)	18.56	18.76	
Hispanic (%)	17.43	15.99	
Stroke Metrics			p-value
Median NIHSS (IQR)	4 (8)	4 (8)	0.2167
Median Onset-to-Arrival Time [minutes (IQR)]	224 (630)	264 (709)	0.9592
Arrive via EMS (%)	58.67	59.19	0.0687
Utilization of IVT (%)	12.92	13.29	0.0647
Utilization of EVT (%)	7.03	8	<0.0001
Median DTP Time [minutes (IQR)]	81 (66)	84 (62)	0.7985
Median DTN Time [minutes (IQR)]	38 (23)	37 (21)	0.0704
Median DTCT Time [minutes (IQR)]	24 (57)	22 (52)	0.3699
DFC Rate (%)	76.27	85.22	<0.0001
Stroke Type			
Acute Ischemic Stroke (AIS) (%)	91.46	92.75	<0.0001
Transient Ischemic Attack (TIA) (%)	8.54	7.25	
