# Supplementary material for: The stroke meta-metric, Defect-Free Care, was maintained year-over-year within the Florida stroke registry during the COVID-19 pandemic
Source: J Stroke Cerebrovasc Dis. Author manuscript; Available in PMC 2025 Feb 1. (PMC11781959; doi:10.1016/j.jstrokecerebrovasdis.2024.108179)
Supplement: MMC3 [file NIHMS2042845-supplement-MMC3.docx]

| Supplemental Table 3: DFC listed by NIHSS severity, Pre-Pandemic (March 2017-February 2020) versus Pandemic (March 2020-February 2021) | | | | |
| --- | --- | --- | --- | --- |
| NIHSS | DFC | Pre-pandemic (n=106,748) | Pandemic  (n=39,845) | P-value |
| Mild (0-5) | Yes | 76.1% | 85.4% | <0.0001 |
|  | No | 23.9% | 14.6% |  |
| Moderate (6-14) | Yes | 79.2% | 86.9% | <0.0001 |
|  | No | 20.8% | 13.1% |  |
| Severe (15-42) | Yes | 84.2% | 88.6% | <0.0001 |
|  | No | 15.8% | 11.4% |  |
