# Supplementary material for: The stroke meta-metric, Defect-Free Care, was maintained year-over-year within the Florida stroke registry during the COVID-19 pandemic
Source: J Stroke Cerebrovasc Dis. Author manuscript; Available in PMC 2025 Feb 1. (PMC11781959; doi:10.1016/j.jstrokecerebrovasdis.2024.108179)
Supplement: MMC1 [file NIHMS2042845-supplement-MMC1.docx]

| Supplemental Table 1: FSR Year-over-Year DFC Rate and the Annual Trends by each of its 7 Domains, 2017-21 (%) | | | | | |
| --- | --- | --- | --- | --- | --- |
| Year | **2017** | **2018** | **2019** | **2020** | **2021** |
| Overall DFC Rate | 74.0 | 76.7 | 83.4 | 86.5 | 87.8 |
| IVT arrive 3.5h, treat by 4.5h | 73.7 | 76.6 | 82.6 | 91.6 | 93.1 |
| Early antithrombotics prescribed | 97.6 | 97.1 | 96.6 | 96.6 | 96.5 |
| VTE prophylaxis prescribed | 97.8 | 97.6 | 97.1 | 96.6 | 96.0 |
| Antithrombotics prescribed | 99.2 | 99.1 | 99.1 | 99.0 | 99.1 |
| Anticoagulation for AFib/Aflutter | 97.1 | 96.3 | 96.9 | 97.1 | 96.4 |
| Smoking cessation documented | 98.1 | 97.9 | 97.3 | 95.3 | 96.6 |
| Intensive Statin prescribed | 97.2 | 97.5 | 97.5 | 97.8 | 98.0 |
