# Supplementary material for: The stroke meta-metric, Defect-Free Care, was maintained year-over-year within the Florida stroke registry during the COVID-19 pandemic
Source: J Stroke Cerebrovasc Dis. Author manuscript; Available in PMC 2025 Feb 1. (PMC11781959; doi:10.1016/j.jstrokecerebrovasdis.2024.108179)
Supplement: MMC2 [file NIHMS2042845-supplement-MMC2.docx]

| Supplemental Table 2: Trends of FSR DFC Rate and its 7 Domains, listed Quarter-by-Quarter (Q1 2017 - Q4 2021) | | | | | | | | |
| --- | --- | --- | --- | --- | --- | --- | --- | --- |
| Quarter | DFC Rate (%) | IVT Arrive 3.5h Treatment 4.5h (%) | Early Antithrombotics (%) | VTE Prophylaxis (%) | Antithrombotics (%) | Anticoagulation for AFib/Aflutter (%) | Smoking Cessation (%) | Intensive Statin (%) |
| 2017Q1 | 73.6 | 73.2 | 97.8 | 98.0 | 99.1 | 97.4 | 98.4 | 96.7 |
| 2017Q2 | 73.2 | 73.8 | 97.5 | 97.9 | 99.1 | 97.1 | 98.4 | 97.2 |
| 2017Q3 | 74.1 | 73.1 | 97.7 | 98.0 | 99.2 | 97.3 | 98.6 | 97.5 |
| 2017Q4 | 75.0 | 74.7 | 97.2 | 97.4 | 99.4 | 96.6 | 97.0 | 97.7 |
| 2018Q1 | 75.8 | 76.6 | 97.1 | 97.5 | 99.2 | 96.8 | 98.5 | 97.6 |
| 2018Q2 | 75.4 | 76.7 | 97.4 | 97.3 | 99.1 | 96.6 | 98.2 | 97.3 |
| 2018Q3 | 76.6 | 75.4 | 97.0 | 97.8 | 99.0 | 96.2 | 97.6 | 97.5 |
| 2018Q4 | 79.2 | 77.8 | 97.2 | 97.7 | 99.0 | 95.6 | 97.4 | 97.7 |
| 2019Q1 | 82.6 | 80.7 | 96.7 | 97.2 | 98.9 | 97.0 | 96.3 | 98.0 |
| 2019Q2 | 83.6 | 80.7 | 97.0 | 97.4 | 99.2 | 97.9 | 97.2 | 97.9 |
| 2019Q3 | 82.6 | 82.0 | 96.7 | 96.9 | 99.1 | 96.6 | 98.1 | 97.6 |
| 2019Q4 | 84.6 | 86.9 | 95.8 | 97.0 | 99.0 | 96.1 | 97.3 | 96.6 |
| 2020Q1 | 86.5 | 91.3 | 96.9 | 97.0 | 99.2 | 97.6 | 96.1 | 97.9 |
| 2020Q2 | 86.9 | 91.6 | 96.4 | 97.4 | 99.1 | 96.5 | 94.6 | 97.9 |
| 2020Q3 | 85.7 | 91.7 | 96.7 | 95.8 | 99.0 | 97.1 | 94.5 | 97.8 |
| 2020Q4 | 86.8 | 92.0 | 96.5 | 96.1 | 98.9 | 97.1 | 96.0 | 97.8 |
| 2021Q1 | 87.5 | 93.6 | 96.8 | 96.7 | 99.1 | 95.6 | 96.3 | 97.9 |
| 2021Q2 | 88.2 | 92.5 | 96.7 | 96.4 | 99.1 | 96.0 | 96.7 | 98.1 |
| 2021Q3 | 87.2 | 93.8 | 96.5 | 95.1 | 99.0 | 97.3 | 96.5 | 97.9 |
| 2021Q4 | 88.3 | 92.5 | 95.9 | 95.9 | 99.3 | 97.0 | 97.1 | 98.0 |
